# Differences in boldness are repeatable and heritable in a long-lived marine predator

**DOI:** 10.1002/ece3.748

**Published:** 2013-10-03

**Authors:** Samantha C Patrick, Anne Charmantier, Henri Weimerskirch

**Affiliations:** 1 Centre d'Etudes Biologiques de Chizé, CNRS-UPR193479360, Villiers-en-Bois, France; 2 Centre d'Ecologie Fonctionnelle et Evolutive, UMR 5175, Campus CNRSCedex 5, 34293, Montpellier, France

**Keywords:** Animal model, Bayesian environment, individual behavioral differences, personality, quantitative genetics, wandering albatross

## Abstract

Animal personalities, composed of axes of consistent individual behaviors, are widely reported and can have important fitness consequences. However, despite theoretical predictions that life-history trade-offs may cause and maintain personality differences, our understanding of the evolutionary ecology of personality remains poor, especially in long-lived species where trade-offs and senescence have been shown to be stronger. Furthermore, although much theoretical and empirical work assumes selection shapes variation in personalities, studies exploring the genetic underpinnings of personality traits are rare. Here we study one standard axis of personality, the shy–bold continuum, in a long-lived marine species, the wandering albatross from Possession Island, Crozet, by measuring the behavioral response to a human approach. Using generalized linear mixed models in a Bayesian framework, we show that boldness is highly repeatable and heritable. We also find strong differences in boldness between breeding colonies, which vary in size and density, suggesting birds are shyer in more dense colonies. These results demonstrate that in this seabird population, boldness is both heritable and repeatable and highlights the potential for ecological and evolutionary processes to shape personality traits in species with varying life-history strategies.

## Introduction

Behavior is often considered to be a plastic trait, changing with time and context (Westeberhard 1989). However, animal personalities, where individuals show consistent behavioral responses, are increasingly being reported, suggesting that limits to plasticity may be adaptive (Dall et al. 2004; Sih et al. 2004). While personality differences have been identified in over 200 species, the mechanism for the evolution and maintenance of such variation is still debated (Dingemanse and Reale 2005; Dingemanse and Wolf 2010; Reale et al. 2010a). Theoretical models predict that life-history trade-offs (LHTs) may drive the evolution of personality differences, as there is a conflict between current and future reproduction (Wolf et al. 2007). These models predict that individuals who have high potential future reproduction should be risk adverse, as they have a lot to lose, whereas those with low reproductive potential will be risk takers, resulting in variation in behavioral strategies (Wolf et al. 2007). These predictions hold both between and within species due to trade-offs between growth and fecundity (Wolf et al. 2007; Biro and Stamps 2008). However, links between LHTs and personality have mainly been documented in short to medium lived species (Biro and Stamps 2008). Recent work suggests that some life-history traits, and subsequent senescence, become more pronounced with increasing longevity (Turbill and Ruf 2010), revealing the possibility that long-lived species may display stronger trade-offs. As such these species may be integral to our understanding of the evolution and maintenance of personality differences.

Although the definition of personality has become increasingly broad over recent decades and now commonly refers to any consistent behavior (Gosling and John 1999; Reale et al. 2010a), much of what we know about animal personality can be attributed to the study of boldness in novel situations, or the shy–bold continuum (Sih et al. 2004). This clearly defined axis of personality, which can be linked to the well-studied structure of human personality (Gosling 2001), can be measured under standardized conditions and allows comparisons between species. The shy–bold continuum offers the opportunity to partition between-individual variation and estimate the inheritance of a personality trait across species.

Seabirds are among the longest lived wild species, and albatrosses regularly reach 50 years of age. They are colonial breeders, nesting in aggregations but are highly vagile, with large foraging ranges (Weimerskirch et al. 1988; Phillips et al. 2007). There are very few studies examining the heritability of traits in seabirds (Boulinier et al. 1997; Barbraud 2000; Peck et al. 2006; Charmantier et al. 2011; Kim et al. 2012), and none examining either broadly defined personality traits, or considering the shy–bold axis of personality. In fact, personality differences in seabirds have rarely been discussed, although there is evidence of individual differences in territory defense, suggesting variation in aggression (Furness 1987; Kazama and Watanuki 2010; Kazama et al. 2012). There is a large body of evidence that seabirds exhibit consistent individual foraging strategies, revealing behavioral and dietary specialization (e.g., Bearhop et al. 2006; Jaeger et al. 2010; Reviewed by Patrick et al. in press), and these can be considered, in the broad sense, to be personality differences. However, many foraging behaviors are widely acknowledged to be strongly influenced by highly variable external forces such as fishing activity (e.g., Bartumeus et al. 2010), and wind and ocean currents (e.g., Weimerskirch et al. 2012; Louzao et al. 2013) making direct comparisons between and within individuals difficult. This makes partitioning behavioral consistency from environmental consistency problematic, and is perhaps why marine biologists have been reticent to call these behaviors “personalities.” By measuring a personality trait at the nest, using a standardized response, we can substantially reduce variation between individual tests, enabling us to compare personality scores within and between individuals.

Using a population of wandering albatross, with a five-generation pedigree (covering 47 years of monitoring), we examine the environmental and genetic components of the shy–bold continuum. Boldness is measured as the response of individuals to a human approacher (Furness 1987), across five breeding seasons, with repeated measures within and between years. From these measures, we extract a boldness score along a standard axis from shy to bold (Gosling 2001; Sih et al. 2004). We consider age, sex, and colony differences in personality and estimate the effects of measurement error across multiple tests and between observers. Using generalized linear mixed models in a Bayesian environment, we estimate the individual repeatability in boldness and combining these data with known pedigree information, we estimate the additive genetic component and associated heritability of this trait. We predict that boldness would be adaptive in this species due to intra- and interspecific interactions (both with avian species and ground predators e.g., rats), at the colony and at-sea, and that this behavior may vary with colony density. Furthermore, as intraspecific variation in life-history traits has been linked to boldness (e.g., Smith and Blumstein 2008; Reale et al. 2010b), this long-lived species is predicted to display individual differences in personality traits.

## Material and Methods

### Data collection

Wandering albatrosses (*Diomeda* exulans) are large, procellariiform seabirds, which breed throughout the subantarctic islands, and forage between the Tropic of Capricorn and the Antarctic continent (Tickell 1968; Weimerskirch et al. 1993). They usually delay breeding until at least 7 years of age (Weimerskirch and Jouventin 1987). The entire population of wandering albatross on Possession Island, Crozet Islands, has been monitored since 1966, with all breeding attempts recorded and chicks ringed. In latest years, approximately 380 pairs bred annually, making the total adult population c. 600 pairs, in this quasibiennial breeding species. Since 2000, less than 5% of individuals caught per year on the island were unbanded (Charmantier et al. 2011), demonstrating the intensive ringing effort and high natal philopatry and recruitment (Inchausti and Weimerskirch 2002). As a result, the pedigree used for the study is based on 5940 individuals of known parentage, over five generations (see Appendix 1 for pedigree details). Although the rate of extra pair paternity in this population is estimated to be approximately 10% (Jouventin et al. 2007), simulations have shown that at this level, with our sample sizes, there is little effect on heritability estimates (Wilson et al. 2003; Charmantier and Reale 2005; Morrissey et al. 2007) and, if anything, our heritability estimates should be underestimated.

### Boldness measures

Between 2009 and 2013 inclusive, the response of individuals to a human approach (from 5 m) has been measured in incubating adults. Tests were carried out when only one pair member was present at the nest, to avoid the confounding effects of mate behavior. The behavioral response was classified on an ordinal scale from zero to five: 0 = No response; 1 = Bird lifts head; 2 = Bird raises up onto tarsus; 3 = Bird vocalizes; 4 = Bird stands up; 5 = Bird leaves the nest. Only one bird left the nest during the tests. The presence of any of these behaviors was recorded to produce a series of scores per individual. For each individual, we used the maximum response recorded during each visit as the measure of “boldness,” as the response to a nonnatural object has been used to place individuals along the shy–bold continuum extensively in the literature (e.g., Furness 1987; Sih et al. 2004). With ordinal measures, there is often concern over the subjectivity in ordering categories. However, if the order represents progressive increase in responsiveness, we predicted that individuals would be more likely to show sequential behaviors. For example, if an individual lifts its head (1) and vocalizes (3), we predicted it will also rise up onto its tarsus (2). 2360 out of 2404 (98%) of observations agreed with this prediction, providing strong support that this ordinal score represents a scale of increasing responsiveness.

### Statistical analysis

#### Factors affecting boldness

We considered the effect of three sources of environmental and experimental variation on boldness scores.

##### Observer

Different researchers carried out the boldness tests, following a brief training period with the observer from the previous year. While two observers carried out tests in multiple years, the biannual breeding of wandering albatross meant that very few birds were tested by two different observers and the remote field site made it impossible to use the same observer regularly. As a result, in this study, year and observer differences are strongly confounded. We fitted observer as a three-level factor, but acknowledge that the cause of this variation is not clear.

##### Observation number

The number of times an individual was tested for boldness was determined randomly by presence on the nest. Each time an observer approached a nest, a boldness score was recorded and birds which by chance were repeatedly present at this time were tested multiple times. If individual birds become habituated to the boldness test, there may result an increase or decrease in responsiveness with sequential tests (e.g., Dingemanse et al. 2012; Stamps et al. 2012). Therefore, we considered the effect of observation number by fitting it as an ordinal factor. This variable was only tested in repeatability models, as heritability estimates were based on the first observation only for all individuals.

##### Colony differences

The population breeds in lose colonies and for this analysis, we grouped these into nine colonies based on previous studies (Milot et al. 2008; Charmantier et al. 2011; Table 1; Appendix 2). Dispersal is relatively low and occurs over short distances on the island (Natal dispersal = 22%; Mean distance = 0.67 km; Charmantier et al. 2011). Colony was fitted as a factor in all models. While colony differences may be considered as biological variation (Wilson 2008), the low dispersal between colonies means that excluding colony difference could artificially inflate heritability estimates. We present models excluding colony effects in the Appendix 3, and results are consistent.

**Table 1:** The difference in boldness between colonies

	Size of breeding population in 2012^1^ (Pairs)	Number of tests	Number of individuals	Mean boldness
Baie du Marin Sud	86	448	221	1.84 ± 0.06
Baie du Marin Nord	54	254	124	1.41 ± 0.08
Plateau Jeannel	35	113	661	1.48 ± 0.12
Crique du Sphinx Sud	31	117	43	1.24 ± 0.11
Crique du Sphinx Nord	18	60	78	1.43 ± 0.16
Crique de la Chaloupe Sud	34	115	75	1.58 ± 0.12
Crique de la Chaloupe Nord	15	43	29	1.53 ± 0.19
Baie Américaine	39	123	93	1.55 ± 0.11
Pointe Basse	359	1131	83	1.04 ± 0.04
Totals	671	2404	1407^2^	

The number of breeding pairs in the last full breeding season (2012) is given, with data on the number of boldness tests carried out. Colonies are listed from South to North (see Appendix 2).

1 The number of breeding pairs is given for 2012 as the 2013 cycle had not been completed at the time to writing.

2 The number of individuals is greater than that given in the text as 13 individuals moved colonies during the study.

We also examined two biological sources of variation, to test factors correlated with boldness differences.

##### Age

For birds born in the population (*N* = 1189), date of ringing was taken as year zero (exact age known) while for individuals ringed as adults (*N* = 167), birds were estimated to have been born 7 years prior to first capture (Weimerskirch and Jouventin 1987). Age was fitted as a continuous variable in models.

##### Sex

All individuals were sexed visually, as the species is sexually dimorphic in size and plumage characteristics (Marchant and Higgins 1990) and this was fitted as a two-level factor in models.

We tested the effect of these five variables on boldness by fitting an ordinal mixed model using the R package: ordinal. Using likelihood ratio tests, we compared models with and without the term of interest. Boldness was fitted as the response variable and individual was included in all models as a random effect.

### Modeling repeatability and heritability

In repeatability and heritability models, we included only significant measurement error, which may lead to bias in the data, and did not attempt to account for biological variables (age or sex; Wilson 2008). Recent work modeling heritabilities in a Bayesian framework has demonstrated robust methods to allow estimations from generalized linear mixed models, maintaining the natural data structure (de Villemereuil et al. 2013). Extending these methods, we used a Bayesian generalized linear mixed model (G_z_LMM), with ordinal data structure.

For all models, to ensure convergence, we combined visual checks with a Heidelberg and Welch Diagnostic (Heidelberger and Welch 1983) and autocorrelation was <0.1 for all models. The posterior distribution was sampled every 200 iterations, following a burn-in period of 20,000 iterations and a general run of 500,000 iterations (1,000,000 for Bayesian ordinal heritabilities). We selected our priors from current recommendations for models (de Villemereuil et al. 2013) and include a sensitivity analysis (Appendix 3) demonstrating that our prior specification has little effect on the results of models.

### Repeatability

For repeatability models, all observations were included for each individual. The repeatability of individual boldness was measured using a Bayesian G_z_LMM, with an ordinal error distribution. We used a parameter expanded prior, with V = 1, ν = 1000, α.μ = 0 and α.V = 1 for the random effects and the residual variance was fixed to 1. The variance of the probit distribution (1) was added to the total population variance.

The variance components were extracted as the mode of the posterior distribution, with 95% credibility intervals given in brackets after estimates and models were run in MCMCglmm (Hadfield 2010). For all repeatability models, the variance explained by individual identity was divided by the total variance in boldness to give a standard estimate of repeatability (Nakagawa and Schielzeth 2010).

### Heritability

We fitted the same basic models to estimate the inheritance of boldness as were used for repeatability estimates. Given the structure of our pedigree (Appendix 1), there were insufficient data to resolve maternal or permanent environment effects. As such, it is not possible to ascertain the influence of these effects on our heritability estimates with our current data. We included pedigree information to allow the population variance to be structured among relatives. The phenotypic (*V*_P_) variance was partitioned into additive (*V*_A_) and cohort effects (birth year [estimated for immigrants]; *V*_C_) by fitting a mixed model with additive genetic and birth year as random effects. Cohort was dropped from all models as it was nonsignificant. Heritability was estimated as *V*_A_/*V*_P_. Heritability was estimated using a univariate animal model with an ordinal structure and the prior specification for repeatability models.

To allow the reader to draw comparisons with basic estimates of heritability, we estimated parent–offspring and sib–sib regression using polychoric correlations (fitted in R package: polycor). As these are polychoric correlations for ordinal data, we are unable to calculate slopes for the heritabilities.

For all heritability models, we extracted the variance components from models run in MCMCglmm (Hadfield 2010) as the mode of the posterior distribution, with 95% credibility intervals given in brackets after estimates. All models were run in R 2.15 (R Development Core Team 2012).

## Results

**Figure 1:**
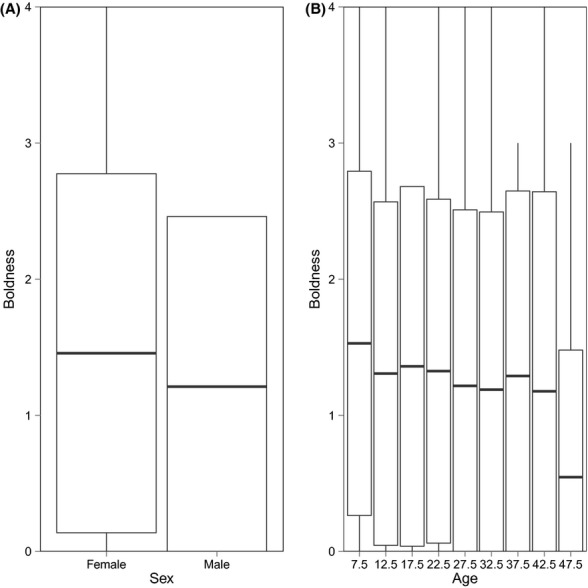
The relationship between age, sex, and personality. (A) The boldness scores of males and females in the population. (B) The difference in boldness with age. For plotting purposes only, the birds are grouped by age into 5-year bins and plotted against the midvalue. In the model, age was fitted as a continuous variable. The mean is shown with a heavy solid line, the box shows ± standard deviation, and solid lines represent the range.

Boldness tests (*n* = 2404) were carried out over four breeding seasons on 1394 birds. 186 tests were carried out in 2009, 254 birds in 2010, 647 birds in 2011, 723 in 2012, and 594 in 2013. Examining repeats within and between breeding seasons, 734 individuals were tested once, 404 twice, 186 three times, 49 individuals four times, 16 individual five times, one six time, two seven times, and one individual eight times. The mean boldness throughout the population was 1.33 ± 0.03. There were observer differences (Likelihood Ratio Test_2_ [LRT_2_] = 19.25; *P* < 0.001) and colony differences (LRT_8_ = 87.41; *P* < 0.001; Table 1), but no effect of observation number (LRT_7_ = 8.28; *P* = 0.31). As a result, observer and colony were included in all repeatability and heritability models. There were sex differences in boldness, such that females were bolder than males (LRT_1_ = 18.73; *P* < 0.001; *F* = 1.46 ± 0.04; *M* = 1.21 ± 0.04; Fig. 1A) and younger birds were bolder than older individuals (LRT_1_ = 9.82; *P* = 0.002; Estimate:−0.02 ± 0.01; Fig. 1B). There was no interaction between colony and age (LRT_8_ = 8.85; *P* = 0.36) nor sex-specific relationships with boldness within colonies (Colony × Sex: LRT_8_ = 5.44; *P* = 0.71).

In the light of previously published studies (Bell et al. 2009), boldness in our population was strongly repeatable (*R*: 0.45 [CI: 0.38–0.51]). Using animal models, we found a boldness to be heritable (*h*^2^ = 0.24 [CI: 0.05–0.41]), with a significant additive genetic component (0.64 [CI: 0.02–1.24]). These results are largely supported by parent–offspring regressions, using raw boldness score, showing moderate heritabilities from mother–offspring (*r* = 0.17, *N* = 306) and sib–sib regressions (*r* = 0.23, *N* = 269), but low heritability from father–offspring regressions (*r* = 0.04, *N* = 276).

## Discussion

In this study, we assay the boldness of nearly 1400 wandering albatrosses, using a simple response to an observer and show that this score is strongly repeatable between individuals. We extend these analyses to show that this trait has a significant additive genetic component and is heritable. Boldness was sex dependent, such that females were bolder than males and we show that boldness appears to decrease with age. There were also colony differences, possibly linked to subcolony size and density. Combined, these results highlight that boldness is an important axis of personality in marine predators and identify behavioral variation that could be the target of selection in a long-lived seabird.

These results improve our understanding of both personality and seabird behavior for two main reasons. First, these estimates of boldness test the response of individuals under similar conditions, allowing direct comparisons between individuals. Most of the early work on animal personality was carried out under standardized conditions, such as in captivity with novel objects or environments (Gosling and John 1999; Sih et al. 2004). This work led to huge advances in our understanding of individual behavior, and enabled us to partition individual responses from environmental variation. While there are many studies examining consistent individual behavior in seabirds (e.g., Bearhop et al. 2006; Jaeger et al. 2010; Reviewed by Patrick et al. [Bibr b41]), these are almost exclusively at-sea measures, where our understanding and ability to quantify the conditions experienced by individuals is poor. This study is one of the first that has measured consistent behavior in seabirds under relatively controlled conditions (but see Kazama and Watanuki 2010; Kazama et al. 2012) and these estimates of boldness could now be linked to at-sea measures of consistent behavior. Individuals vary in their association with fisheries (Votier et al. 2010; Torres et al. 2011; Granadeiro et al. 2013, 2011) and the distance they forage from the colony (Reviewed by Patrick et al. in press), both of which are thought to be linked to competitive ability, and as such boldness may predict an individual's foraging strategy.

Second, we demonstrate that boldness is heritable in a very long-lived species. This is one of only a handful of studies to demonstrate heritable behavior in seabirds (See also Boulinier et al. 1997; Barbraud 2000; Peck et al. 2006; Charmantier et al. 2011; Kim et al. 2012) and the first to show the genetic origin in variation of personality. Trade-offs with current and future reproduction, such as senescence, have been shown to increase with longevity (Turbill and Ruf 2010), and here we show that personality differences are repeatable and heritable in one such long-lived species. This paves the way for studies examining the implications of personality differences in individual life-history strategies in long-lived species, and offers a possible system, with both high individual variation in life-history strategies and repeatable and heritable personality differences, to examine the evolution and maintenance of personality variation. Specifically, there is widely available information of foraging behavior, parental investment, and intraspecific competition, all of which may be tightly linked to boldness and integral to the evolution and maintenance of personality variation.

The structure of our pedigree prohibited us from partitioning the common parental environment effects from additive genetic variation. This can lead to an overestimation of heritability as similar phenotypes may emerge as a result of rearing environment (Kruuk and Hadfield 2007). Four of the five studies measuring maternal effects for personality traits in the wild have found no evidence that they explain a substantial proportion of variation (Sinn et al. 2006; Duckworth and Kruuk 2009; Reale et al. 2009; Blumstein et al. 2010), but the most recent study found significant maternal effects for three personality traits in red squirrels (Taylor et al. 2012). It has been suggested that maternal effects may be particularly strong in species with extended offspring care (Reinhold 2002). As wandering albatrosses show extremely prolonged offspring care, rearing chicks across a 13-month period, attempts should be made to quantify maternal environmental and genetic effects when the data become available.

In this study, we show links between an axis of personality and three sources of variation. The boldness of individuals was markedly different between the colonies. As dispersal is low between sites (Charmantier et al. 2011; Appendix 2), differences in boldness could emerge if directional selection and mating patterns differed at the colony level, resulting in divergence in boldness. The Southern colonies (all excluding Pointe Basse) are a series of small subcolonies, divided by valleys, where birds breed at low densities. Birds from Pointe Basse, where individuals breed at high densities, were considerably shyer, suggesting that boldness may be associated with low-density nest sites. However, as we have only one high-density colony, we cannot be certain that other factors do not influence boldness in addition to density. To disentangle these effects, several other high-density colonies need to be studied or individual nest density at Pointe Basse in relation to boldness. It has also been shown in this population that birds are more likely to disperse to smaller colonies with more available nest sites (Gauthier et al. 2010) and previous work has shown that bolder birds are more likely to disperse (Cote et al. 2011; Quinn et al. 2011). As a result, the pattern we see here may be as a result of historic movements by bolder birds, dispersing to smaller colonies. There are also heterogeneous interactions with humans between colonies, and as a result Baie du Marin Sud and Pointe Basse have greater human presence. However, in this study, these are the boldest and shyest colonies, suggesting that human interaction is not driving differences in boldness. However, these colonies also differ in size and as such future work should examine boldness in large colonies with no human presence.

We also found that females were bolder than males, supporting extensive work detailing sex differences in personality (Reviewed by Schuett et al. 2010). While the direction varies between species, females regularly exhibit bolder behaviors and it is suggested that sexual selection may drive these differences (Schuett et al. 2010). Sex-specific behaviors are widely reported in seabirds (Lewis et al. 2002) and in this species, sex difference has been found in physiology (Angelier et al. 2007), growth rates (Weimerskirch and Lys 2000), diet (Ceia et al. 2012), and foraging behaviors (Weimerskirch and Jouventin 1987). As personality differences have previously been linked to physiology and foraging behaviors (Sih et al. 2004; Reale et al. 2010a), we suggest that sex differences in boldness could result in, or occur as a result of, different strategies between the sexes.

We also report that older birds appeared to be shyer than younger individuals. This result opposes most theoretical predictions, which suggest that older individuals should be bolder, as they are predicted to invest more in current reproduction (Wolf et al. 2007; Biro and Stamps 2008). However, these effects may be attenuated in long-lived species, and the evidence of senescence in albatross, such that older individuals have lower reproductive success (Lecomte et al. 2010), may explain decreasing boldness with age. Alternatively, selection may act on personality and survival, and as a result the effects we observe may be a result of selective disappearance of bolder individuals with increasing age. At the other end of the age continuum, highly variable age at first reproduction could account for differences if bolder individuals attempt to breed earlier, but this does not appear to occur in our population (S.C Patrick and H. Weimerskirch, unpubl. data).

State-dependent differences in personality are integral to the majority of models predicting the persistence of personality variation (Dingemanse and Wolf 2010; Wolf and Weissing 2010). While in the past, many personality studies attempt to quantify behaviors which are static over time, recent work has highlighted the importance of within-individual changes in personality traits (Dingemanse et al. 2010). Long-lived seabirds such as albatrosses, which are well known for plasticity in other behaviors (Gremillet and Boulinier 2009), would be an ideal system to model these within-individual changes in personality over time. By assaying individuals over longer temporal periods and different life stages, individual trajectories could be measured and aspects of plasticity in personality quantified. Furthermore, this type of study could assess whether sex differences emerge at birth, or develop due to selection through adulthood and could attempt to ascertain the causes of colony differences in personality. Measures of selection acting between colonies could help assess whether local adaptation is shaping colony structure and help allude to the evolutionary potential of this heritable trait. In conclusion, we show that the wandering albatross has a highly repeatable and heritable boldness, quantified along the shy–bold axis of personality. Furthermore, we demonstrate that these differences are tightly linked to ecological and state-dependent variation. This reveals the evolutionary and ecological importance of boldness in this species, and its extreme life-history strategy offers a huge potential to investigate plasticity and state-dependent changes in personality.

## Supporting Information

**Appendix 1.** Summary pedigree details.

**Appendix 2.** The rate of dispersal between colonies.

**Appendix 3.** Additional analyses: sensitivity analysis and animal models including permanent environment effects.
