# Supplementary material for: Differences in boldness are repeatable and heritable in a long-lived marine predator
Source: Ecol Evol. 2013 Oct 3;3(13):4291–9. doi: 10.1002/ece3.748 (PMC3856731; doi:10.1002/ece3.748)
Supplement: Supplementary file 1 [file ece30003-4291-SD1.docx]

**Appendix 1**

Summary statistics from the pedigree used in all models (Extracted using R package: pedantics; Morrissey & Wilson 2010)

| Number of records | 2208 |  |  |  |
| --- | --- | --- | --- | --- |
| Number of founders | 1348 |  |  |  |
| Number of full sibs | 292 |  |  |  |
| Non-zero F | 0.00 |  |  |  |
| F > 0.125 | 0.00 |  |  |  |
| Mean pairwise relatedness | 0.0005 |  |  |  |
| Pairwise relatedness>=0.125 | 0.0012 |  |  |  |
| Pairwise relatedness>=0.25 | 0.0011 |  |  |  |
| Pairwise relatedness>=0.5 | 0.0008 |  |  |  |
| **Maternal Summary** |  |  |  |  |
| Number of maternities | 860 |  |  |  |
| Number of maternal sibs | 355 |  |  |  |
| Number of maternal grandmothers/ fathers | 140 |  |  |  |
| Mean maternal sibship size | 1.42 |  |  |  |
| **Paternal Summary** |  |  |  |  |
| Number of paternities | 860 |  |  |  |
| Number of paternal sibs | 371 |  |  |  |
| Number of paternal grand mothers/ fathers | 117 |  |  |  |
| Mean paternal sibship size | 1.44 |  |  |  |

**Appendix 2**

Map of the subcolonies on Possession Island, Crozet. Adapted from Charmantier et al (2011). Plateau Jeannel Colony added from previously published maps.


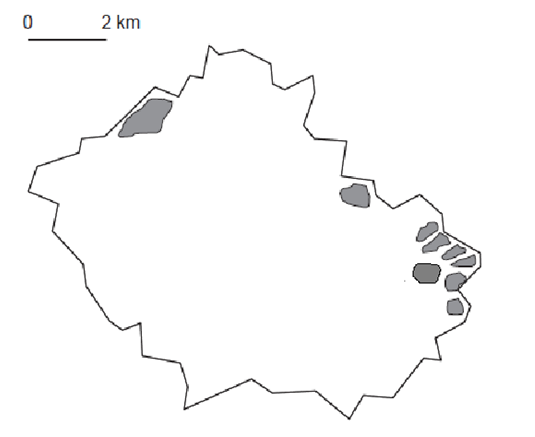


Pointe Basse

Baie Américaine

Plateau Jeannel

Crique de la Chaloupe

Crique du Sphinx

Baie du Marin

N

N

S

S

N

S

51.7

51.8

51.9

-46.35

-46.40

-46.45

Table S1: The dispersal between subcolonies and degree of natal philopatry

|  | **Breeding Colony** | | | | | | | | | | |  |
| --- | --- | --- | --- | --- | --- | --- | --- | --- | --- | --- | --- | --- |
| **Birth Colony** | Baie du Marin Sud | Baie du Marin Nord | | Plateau Jeannel | | Crique du Sphinx Sud | Crique du Sphinx Nord | Crique de la Chaloupe Sud | Crique de la Chaloupe Nord | Baie Américaine | Pointe Basse | Natal Philopatry (%) |
| Baie du Marin Sud | **253** | | 44 | | 4 | 5 | 1 | 2 | 0 | 1 | 4 | 81 |
| Baie du Marin Nord | 34 | | **133** | | 18 | 7 | 2 | 3 | 4 | 4 | 4 | 64 |
| Plateau Jeannel | 4 | | 3 | | **24** | 3 | 1 | 0 | 2 | 0 | 0 | 65 |
| Crique du Sphinx Sud | 8 | | 11 | | 18 | **52** | 11 | 5 | 2 | 2 | 0 | 48 |
| Crique du Sphinx Nord | 1 | | 0 | | 3 | 4 | **12** | 10 | 2 | 0 | 0 | 38 |
| Crique de la Chaloupe Sud | 1 | | 2 | | 4 | 8 | 9 | **51** | 3 | 3 | 3 | 64 |
| Crique de la Chaloupe Nord | 4 | | 1 | | 3 | 3 | 5 | 10 | **22** | 2 | 0 | 44 |
| Baie Américaine | 1 | | 1 | | 4 | 0 | 3 | 1 | 1 | **66** | 8 | 78 |
| Pointe Basse | 6 | | 1 | | 2 | 0 | 0 | 0 | 0 | 3 | **757** | 98 |

**Appendix 3**

**Additional heritability analyses**

**Table S2: The effect of colony on estimates of heritability. Models were rerun excluding colony as a fixed effect.**

| **Estimate** | **Error structure** | **Subset** | **Fixed effects** | **Estimate** |
| --- | --- | --- | --- | --- |
| Repeatability | Ordinal | All Data | Observer | 0.48 (0.42-0.53) |
|  |  |  |  |  |

**Sensitivity analysis**

**Table S3: The impact of prior specification on estimates of heritability and repeatability**

| **Estimate** | **Error structure** | **Prior** | **Fixed effects** | **Estimate** | **V_A_** | **V_P_** |
| --- | --- | --- | --- | --- | --- | --- |
| Repeatability | Ordinal | Inverse Gamma | Observer + Colony | 0.45 (0.38-0.51) | NA | NA |
| Repeatability | Ordinal | Informative | Observer + Colony | 0.47 (0.39-0.51) | NA | NA |
|  |  |  |  |  |  |  |
| Heritability | Ordinal | Inverse Gamma | Observer + Colony | 0.21 (0.00-0.38) | 0.39 (0.00-1.22) | 1.39 |
| Heritability | Ordinal | Informative | Observer + Colony | 0.22 (0.01-0.36) | 0.35 (0.02-1.11) | 1.35 |

Prior specifications: Inverse Gamma: R = (V = 1, nu = 0.002), G = (V = 1, nu = 0.002); Informative Prior: R = (V = matrix(var(Personality),* 0.05), nu = 1), G = (V = matrix(var(Personality)* 0.05),nu = 1)). For all ordinal models the residual variance was constrained to 1 (V = 1, fix = 1).

**Reference List**

Charmantier, A., M. Buoro, O. Gimenez and H. Weimerskirch. 2011. Heritability of short-scale natal dispersal in a large-scale foraging bird, the wandering albatross. *Journal of Evolutionary Biology,* 24: 1487-1496.

Morrissey, M. B. and A. J. Wilson. 2010. Pedantics: an r package for pedigree-based genetic simulation and pedigree manipulation, characterization and viewing. *Molecular Ecology Resources,* 10: 711-719.
